# Morphology control of zinc oxide films via polysaccharide-mediated, low temperature, chemical bath deposition

**DOI:** 10.3762/bjnano.6.83

**Published:** 2015-03-24

**Authors:** Florian Waltz, Hans-Christoph Schwarz, Andreas M Schneider, Stefanie Eiden, Peter Behrens

**Affiliations:** 1 Institut für Anorganische Chemie, Leibniz Universität Hannover, Callinstraße 9, 30167 Hannover, Germany; 2 Bayer Technology Services GmbH, 51368 Leverkusen, Germany

**Keywords:** chemical bath deposition, hyaluronic acid, polysaccharide, transparent conductive oxide, zinc oxide

## Abstract

In this study we present a three-step process for the low-temperature chemical bath deposition of crystalline ZnO films on glass substrates. The process consists of a seeding step followed by two chemical bath deposition steps. In the second step (the first of the two bath deposition steps), a natural polysaccharide, namely hyaluronic acid, is used to manipulate the morphology of the films. Previous experiments revealed a strong influence of this polysaccharide on the formation of zinc oxide crystallites. The present work aims to transfer this gained knowledge to the formation of zinc oxide films. The influence of hyaluronic acid and the time of its addition on the morphology of the resulting ZnO film were investigated. By meticulous adjustment of the parameters in this step, the film morphology can be tailored to provide an optimal growth platform for the third step (a subsequent chemical bath deposition step). In this step, the film is covered by a dense layer of ZnO. This optimized procedure leads to ZnO films with a very high electrical conductivity, opening up interesting possibilities for applications of such films. The films were characterized by means of electron microscopy, X-ray diffraction and measurements of the electrical conductivity.

## Introduction

Zinc oxide is a unique material with a number of interesting properties such as piezo- and pyro-electricity [1–2], high optical transparency [3], catalytic activity [4–5], and chemical sensing [6–8]. It is also one of the most promising candidates for the replacement of indium tin oxide (ITO) in transparent conductive oxide (TCO) applications [9–10]. Hence, ZnO films are a key research area in industry as well as in academia with more than 2100 publications in 2013 (Thomson Reuters, Web of Knowledge). Several methods have been used to deposit ZnO on different substrates, for example, pulsed laser deposition (PLD) [11], chemical vapor deposition (CVD) [12–13], as well as wet chemical approaches such as sol–gel synthesis [14] and chemical bath deposition (CBD) [15–18]. Among these, CBD methods have gained increasing interest since they allow the deposition of ZnO films in large-scale applications at low temperature, on a number of different substrates and with minimal effort.

ZnO is a semiconducting, ceramic material with a direct band gap of 3.37 eV and an exciton binding energy of 60 meV [19]. Although ZnO is reported to be an n-type semiconductor (most likely due to the hydrogen impurities which act as shallow donors), it is a challenging task to control its conductivity [20]. In general, in applications where highly conductive materials are required (e.g., solar cells and light emitting diodes (LEDs)), ZnO must be doped.

Several groups have reported the successful doping of ZnO films with dopants such as magnesium [21], iodine [22], boron [23–24], titanium [25], manganese [26], and aluminium [27–29]. These films were grown via CBD or related techniques (e.g., double dipping or hot water dipping). In CBD processes, hexamethylenetetramine (HMTA) is usually dissolved in a solution containing Zn(II) ions. At a certain temperature, HMTA decomposes and consequently delivers hydroxide ions, forcing the formation of crystalline ZnO [30]. Doping is carried out by the simple addition of the corresponding dopant salt to the deposition solution. In addition to doping, the microstructure of the resulting film, which involves the crystallite size as well as the morphology of the crystallites and the degree of their intergrowth, has a decisive influence on many applications, for example, in sensors and catalysts [8,31].

As the wurtzite structure of ZnO is polar, crystals of the substance feature two differently charged surfaces: the oxygen terminated (00−1) and the zinc terminated (001) faces, on both of which charged molecules can be chemisorbed by electrostatic interactions. In addition, the uncharged {100} faces of ZnO can support the physisorption of molecules. Such adsorption phenomena can influence the growth rates of the corresponding faces, leading to different crystal habits.

**Figure 1:**
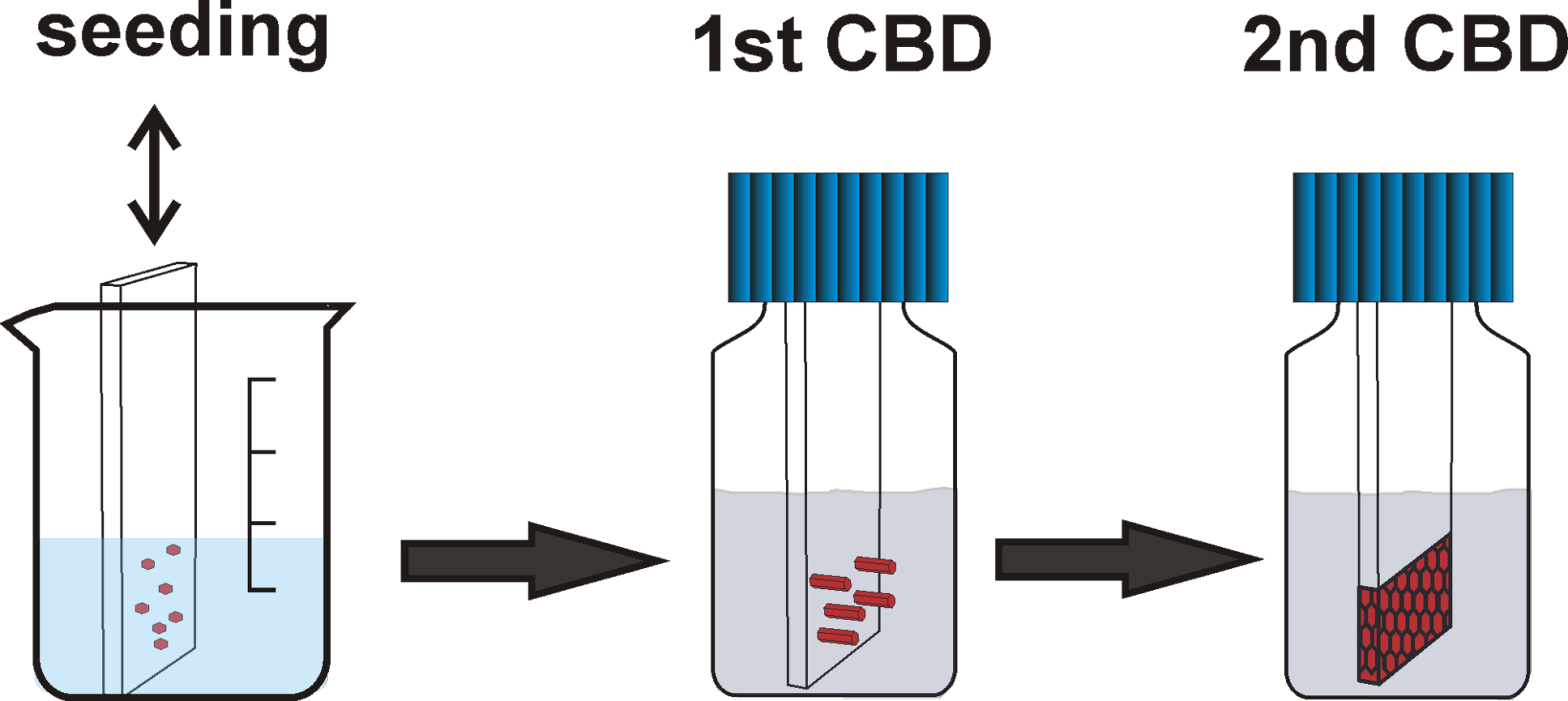
Scheme of the three-step, ZnO film deposition process. Seeds were deposited on glass slides by immersion in a Zn(II) solution, followed by annealing. In the first CBD step, different ZnO morphologies can be grown depending on the time of the HYA addition. In the second CBD step, a dense film can be formed.

Solvent-based chemical deposition processes are particularly suited for the addition of molecules that may affect the morphology of ZnO crystals and their aggregates as well as of ZnO films. Molecules such as citrate [31–32], histidine [33], 1-butan-2-ylpyrrolidin-2-one (PVP) [34–35], 2-hydroxybutanedioate (malate) [36], ascorbate [37], diaminopropane [38], hexadecyl(trimethyl)azanium bromide (CTAB) [39], and block copolymers [40] have been used for this purpose, in addition to naturally occurring amino acids and peptides [41], which have already been successfully applied in this respect. We recently investigated the influence of two polysaccharides, hyaluronic acid (HYA) and chondroitin-6 sulfate (C6S), on the morphology of primary ZnO crystallites and on their aggregates, as they are formed in precipitation experiments [42]. Whereas C6S leads to a pronounced platelet-like morphoplogy of the primary crystallites, HYA leads to the growth of small wedge-like particles and the aggregation of these particles into bundles. We surmised that this influence of HYA might be beneficial to the quality of deposited, thin, ZnO films by increasing the number of primary crystallites. This should lead to finer structured films with more strongly intergrown crystals, thus enhancing the electrical conductivity and optical transparency. Therefore, we have undertaken the study presented here, where ZnO films were prepared in a three-step process: a seeding step, followed by two CBD steps (Figure 1). In the first of the two CBD steps, HYA was added at different time intervals in order to optimize the quality of the resulting films. The properties of the films were studied by means of field emitting scanning electron microscopy (FE-SEM), X-ray diffraction (XRD), UV–vis spectroscopy and electrical conductivity measurements.

## Results and Discussion

ZnO films were prepared according to the three-step process described in the Experimental section and depicted in Figure 1.

### Step 1: Seeding

The solution-based growth of zincite in general requires prior application of crystalline seeds on the support. In our work, the solution deposition procedure according to Greene et al. reproducibly led to high film quality in the final product [43]. The seeding did not result in clouding of the glass slides, which would have been observable with the naked eye. The XRD patterns of glass slides seeded in this way displayed only a broad signal originating from the amorphous glass (data not shown). FE-SEM also failed to visualize the seeds on the glass slides, probably due to their small size and the strong electric charging of the substrate.

However, indirect evidence of a successful seeding was possible. Contact angle measurements showed that the slides are slightly more hydrophobic after the seeding process. The contact angle of a seeded glass slide was about 58° in comparison to 46° for a clean glass slide. Furthermore, the UV–vis spectra of seeded glass slides showed an absorption band in the UV range at approximately the energy of the ZnO band gap (3.37 eV) (data not shown). However, the final evidence is presented by the efficient growth of ZnO on the seeded slides; in contrast, unseeded slides did not properly support the growth of ZnO.

### Step 2: First CBD

**Figure 2:**
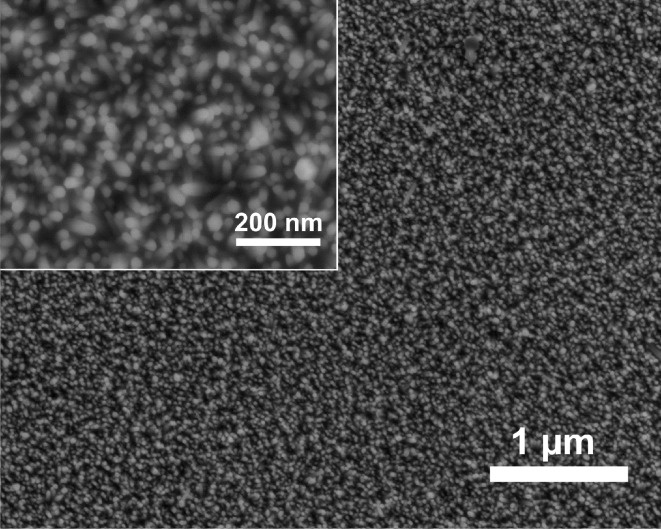
SEM micrographs of a ZnO nanorod array grown on a seeded glass slide for 1 h without the addition of HYA; the inset shows a higher magnification image.

**Figure 3:**
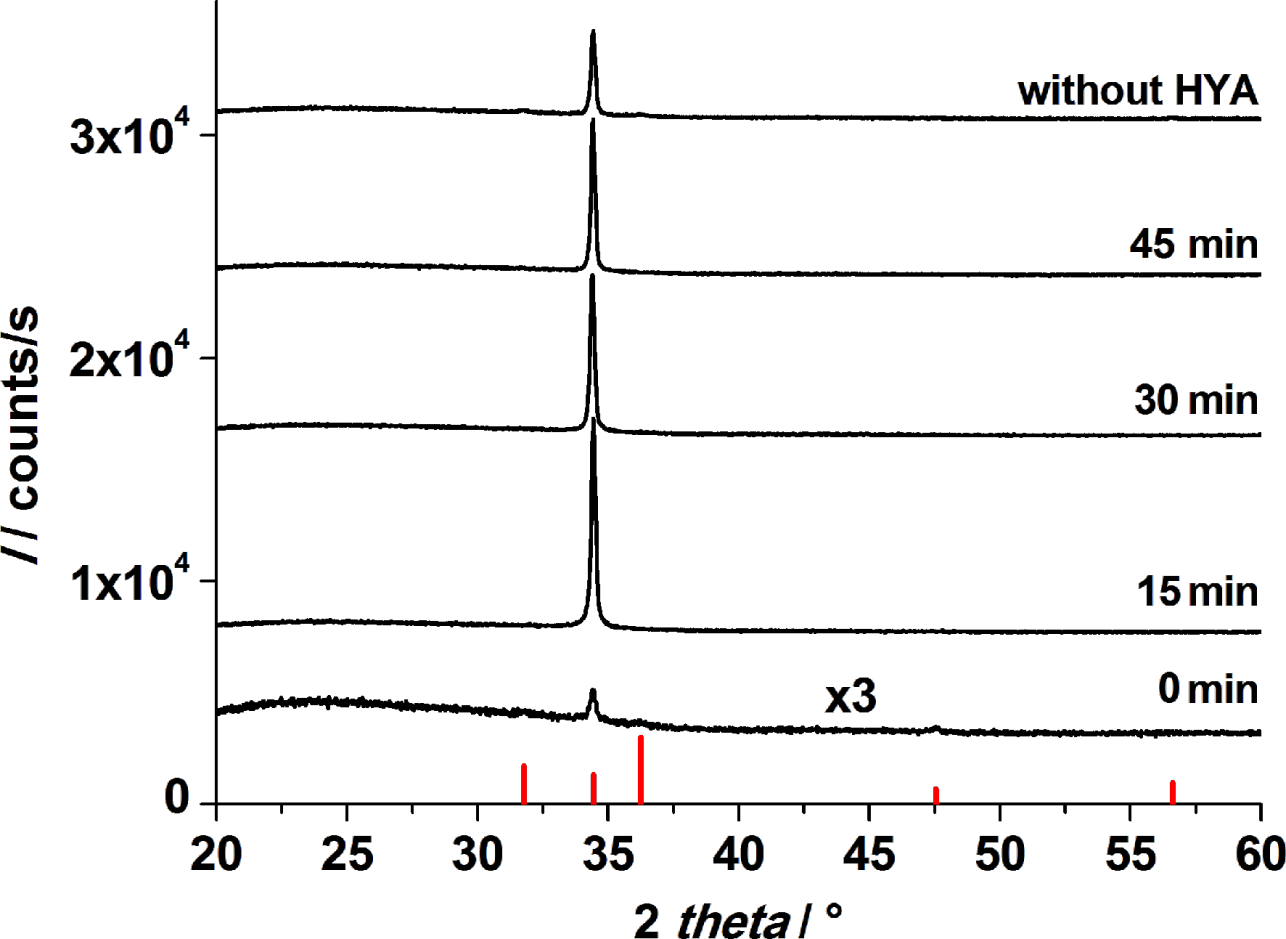
X-ray diffraction patterns of ZnO films after the first CBD. Growth was performed for 1 h in total with and without the addition of HYA. HYA was added after different time intervals as indicated in the figure. The red bars mark the XRD reflection peaks from a zincite reference [45].

In the absence of hyaluronic acid (HYA), highly vertically aligned ZnO nanorods grow on priorly seeded glass slides, when the procedure described in the Experimental section is applied. The growth of aligned ZnO nanorods arrays on different substrates has been previously reported [15,17,43–44]. The scanning electron micrographs in Figure 2 show a nanorod array that was grown for 1 h. In X-ray diffraction experiments, arrays of this kind display only the (002) reflection of zincite due to the strong texture of the crystals with their *c* axis perpendicular to the support (Figure 3).

As previously demonstrated, the addition of natural polysaccharides affects the morphology of the ZnO crystallites precipitated from solution [42,46–47]. This occurs largely due to the blocking of specific crystal faces during growth. In precipitation experiments, performed under conditions that are very similar in concentration and temperature to typical ZnO CBD processes, the addition of HYA led to the formation of well-defined and highly symmetric ZnO mesocrystals. Using this procedure, the size of the individual ZnO particles was dramatically decreased from the micrometer down to the nanometer scale [42].

In order to investigate the influence of HYA on the morphology of the resulting zinc oxide, HYA was dissolved in water during the first CBD at different time intervals (0, 15, 30 and 45 min). The growth of the ZnO nanorods (Figure 2) is assumed to proceed continuously on the seeded glass slides until HYA is added to the reaction mixture, which at this point may affect the further deposition and growth of ZnO.

The XRD patterns of films obtained after the first CBD (Figure 3) display only the (002) reflection of zincite, irrespective of whether HYA was supplied or not. This finding evidences the perpendicular alignment of the *c* axis of the ZnO crystallites with respect to the glass surface, which is unaffected by the addition of HYA. However, the intensity of the (002) reflection is very weak for the film grown when HYA was immediately added, indicating a strong decrease in the deposited amount of ZnO for this case. When HYA was added to the solution at a later point in time (15, 30 or 45 min), the (002) signal was more intense, indicating that more ZnO was grown on the substrate. These findings agree with the assumption that the presence of HYA decreases the ZnO deposition rate, for example by blocking the growth of certain crystal faces. Curiously, the sample prepared without the addition of HYA displays a weaker signal than samples with HYA added after 15, 30 and 45 min. This finding will be further discussed with regard to SEM investigations.

**Figure 4:**
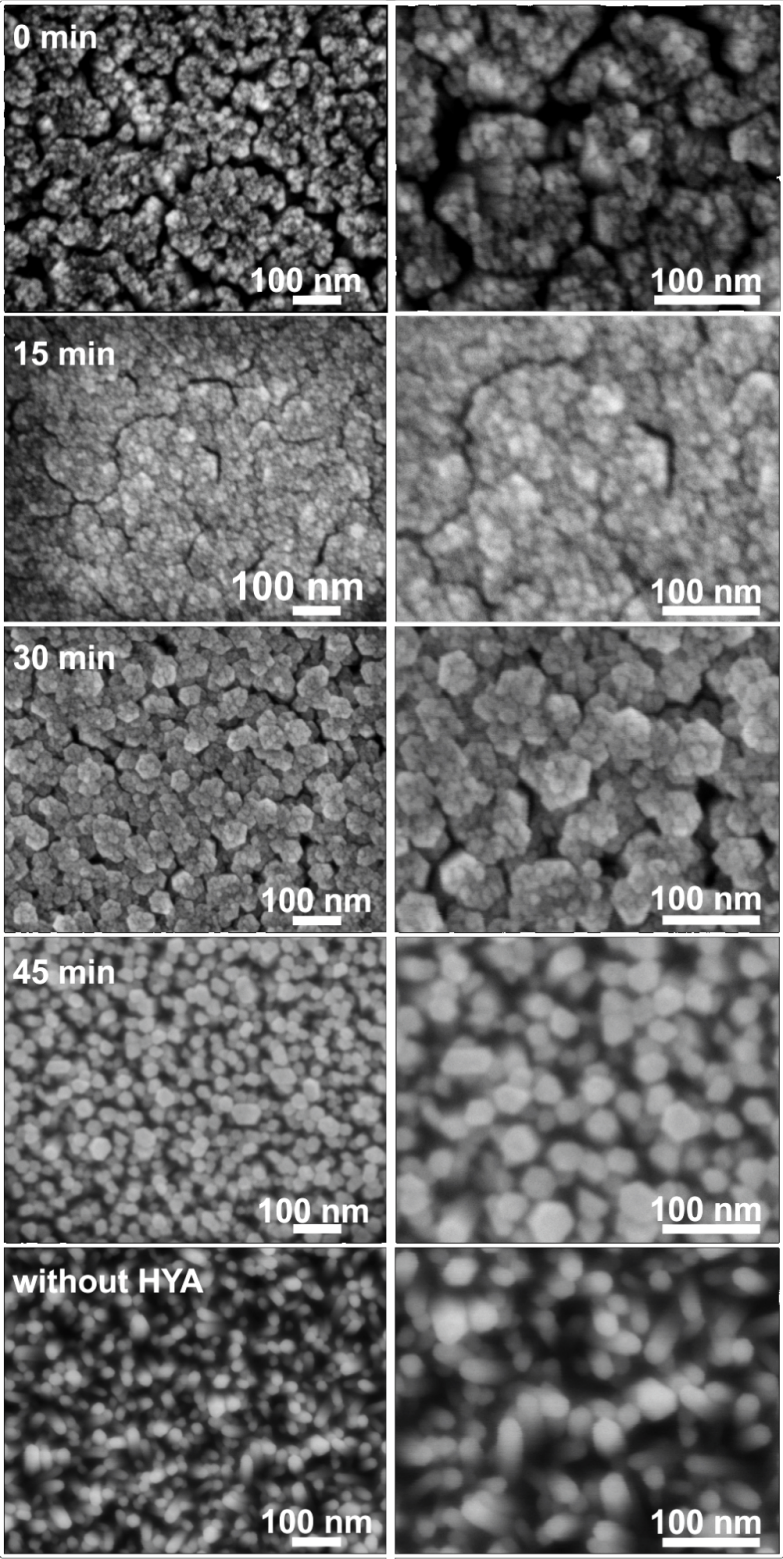
SEM micrographs of ZnO films after the first CBD. Growth was performed for 1 h in total with and without the addition of HYA. HYA was supplied after specific time intervals during the growth process as indicated.

Whereas the crystallographic orientation of the ZnO crystallites on the support is not affected by the addition of HYA, the ZnO film morphology changes dramatically when the CBD is performed in the presence of HYA. This is exhibited in SEM micrographs, which provide views of the plane of the deposited films (Figure 4). In general, the diameter of the individual ZnO nanorods decreased strongly when the HYA was added within the first 30 min of reaction, specifically, much finer structures were obtained. However, the individual nanocrystallites aligned themselves to larger aggregates, and the deposits can better be described as bundles of needles rather than as individual nanorods. This is comparable to precipitation experiments in which HYA adsorbs onto ZnO crystallites during their growth and thereby influences their size and aspect ratio. Furthermore, those ZnO subunits aggregate under the influence of HYA into highly ordered mesocrystals, which was evidenced by SEM investigations and selected area electron diffraction [42]. In the film deposition experiments described here, these aggregates even display a common hexagonal morphology, which can for example be seen in Figure 4 on the product prepared with HYA addition after 30 min. When HYA is added only after 45 min of reaction time, it has no significant influence on the morphology of the film. In fact, the SEM image of this sample is similar to that of the sample prepared without HYA addition. We assume that the zinc ions have already been almost completely consumed after this reaction time and that growth had completed before HYA addition.

With regard to the increasing intensity of the (002) reflections in the XRD patterns of the films after 15, 30 and 45 minutes of reaction, the SEM micrographs indicate that this increase is due to an increased lateral growth of the ZnO crystallites, which confirms that more ZnO was deposited when HYA was added at a later point in time. On the contrary, the sample in which HYA was immediately added shows also a very dense lateral growth, whereas the XRD reflection intensity is very weak. Therefore, we assume that the axial growth perpendicular to the support is inhibited by the immediate addition of HYA, leading to a lower mass of ZnO and consequently to a less intense signal in the XRD pattern. This assumption will be further discussed by support of cross-section SEM investigations presented in the next section.

During the first CBD, the morphology of ZnO grown on the seeded glass slides can be tailored by the addition of HYA: When no HYA is added or when it is added only after 45 min, arrays of individual nanorods are formed. When HYA is immediately added or up to a reaction time of 30 min, finely structured bundles of needle-shaped ZnO crystals are observed. Since the crystalline domains of these small crystallites do not overlap very well after the first CBD growth step, the electrical conductivity is only moderate. The sheet resistance of the films after the first CBD is typically in the range of MΩ/sq. Therefore, an additional step is necessary to grow a dense, ZnO film in order to yield low electrical resistance for the final sample.

### Step 3: Second CBD

**Figure 5:**
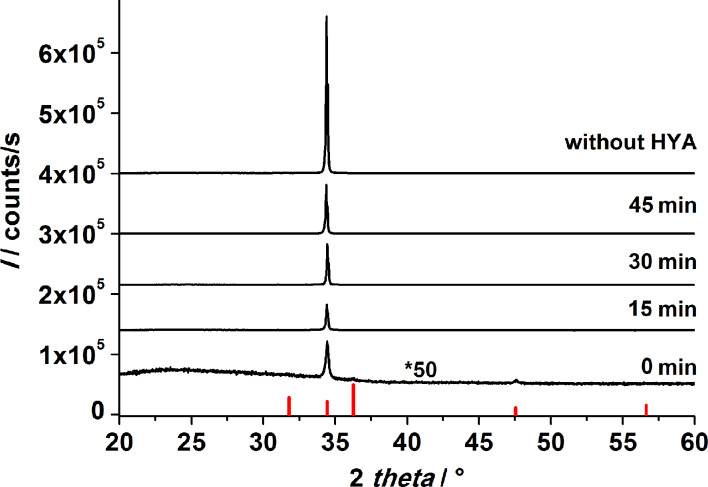
X-ray diffraction patterns of ZnO films after the second CBD. The films differ in the addition time of the HYA in the first CBD step (from 0 min up to 45 min). The red lines represent the XRD reflection peaks from a zincite reference [45].

The reaction conditions for the final growth step were adopted from Baxter and Schmuttenmaer, who obtained intergrown ZnO films after a reaction time of 3 h [48]. In our experiments, the reaction time could be reduced to 1 h due to the excellent growth conditions provided by the substrate during the first CBD step. The XRD patterns recorded after this third step show only (002) reflections (Figure 5), irrespective of the details of the first CBD step, proving that the growth of ZnO continues to proceed with the *c* axis perpendicular to the support.

In general, the intensity of the (002) signal strongly increased after the third step as compared with the signals obtained on samples after the first CBD. This further indicates the successful deposition of ZnO. However, the intensity of this peak differs between the samples after the third step:

- The film which was grown in the first CBD step with immediate HYA addition shows the weakest signal, suggesting that this film supports further ZnO growth the least.
- The samples prepared in the first CBD step with HYA addition over the time intervals between 15 to 45 min showed a slight increase in the intensity of the (002) signal after the third step. The increase was stronger when HYA was added later.
- The film which was prepared with no HYA addition showed a 3× higher signal than films grown with HYA in the first CBD, indicating that a higher amount of ZnO was deposited.

**Figure 6:**
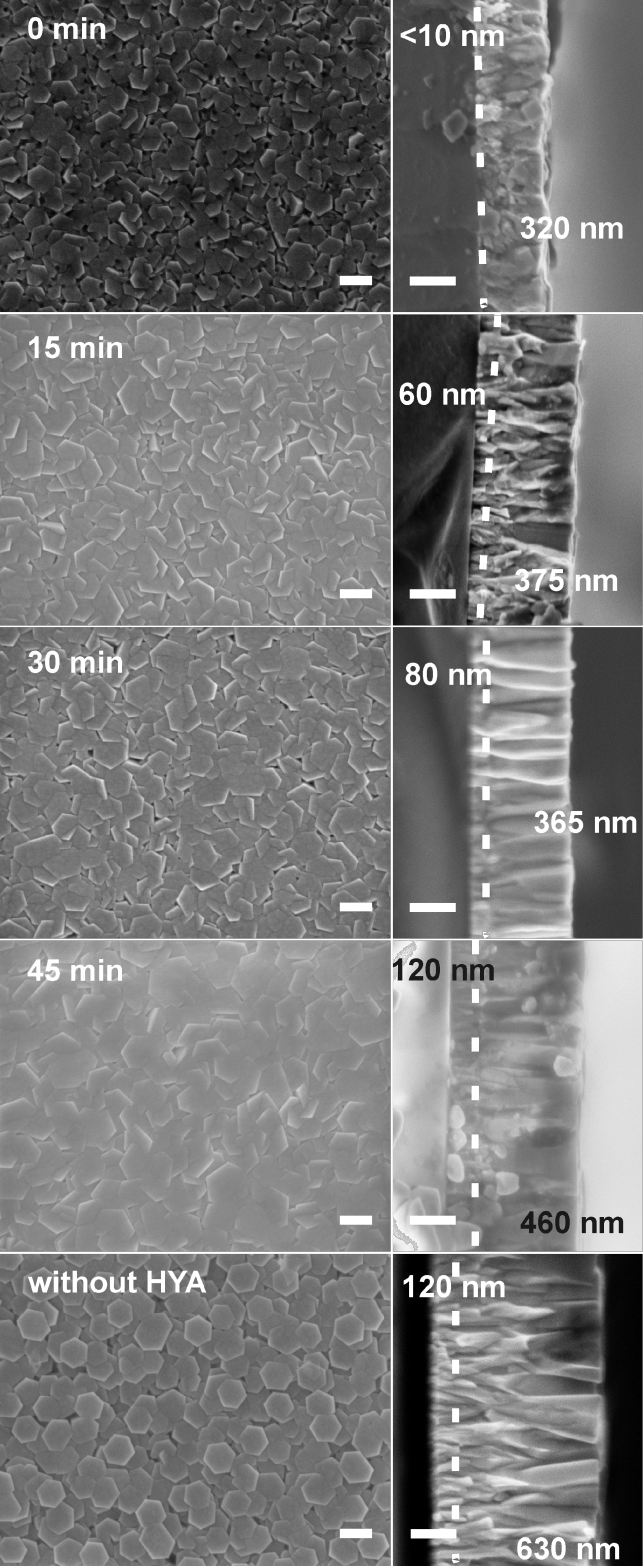
SEM micrographs in plan view (left) and corresponding cross sections (right) of ZnO films after the second CBD step. The films were prepared both without HYA and with different addition times of HYA during the first CBD step (scale bars: 200 nm). The dotted lines indicate the interface between the ZnO grown in the first and the second CBD steps. The values in nm correspond to the film thicknesses of the ZnO grown within the first (to the left of the dotted line) and the second CBD (to the right of the dotted line) steps, respectively.

Obviously, not only the growth of the films in the first CBD step is affected by the HYA addition, but also the growth rate in the second CBD step is strongly influenced. The film morphology after the second CBD step determines the final properties of the films. Figure 6 displays SEM micrographs of these films taken in plan view and as cross sections. All films show hexagonal poles oriented perpendicular to the support with lateral sizes in the range of 200 nm. However, they differ strongly in the degree of intergrowth, depending upon the addition time of HYA during the first CBD. Whereas the films prepared with HYA exhibit highly intergrown crystallites (among these, the effect is weakest for the film prepared with an addition time of 30 min), the crystallites on the film prepared without HYA display a much weaker crystallite intergrowth.

The corresponding cross section SEM micrographs of the ZnO films confirm the results of the XRD analysis. The addition of HYA during the first CBD step affects the amount of ZnO deposited during the second CBD step. The maximum overall film thickness was achieved without addition of HYA, where the film grew to a thickness of approximately 750 nm. The earlier the HYA was added during the first CBD, the more the perpendicular growth was inhibited in the second CBD step. Thus, the thinnest films (approximately 320 nm) are obtained by immediate addition of HYA. The film thickness consecutively increased in a nonlinear manner from 435 nm, 460 nm to 590 nm for addition times of 15, 30 and 45 min, respectively.

The closer inspection of the cross section SEM images of the films (micrographs on the right of Figure 6) reveals further details of their morphology. A rod-like morphology can be assigned to zincite crystallites deposited during the first CBD step, whereas a more branched growth has obviously occurred during the second CBD (these two regions are separated by dotted lines in the micrographs in Figure 6). The individual film thicknesses taken from the cross section SEM micrographs are compiled in Table 1.

**Table 1:** Individual film thicknesses of ZnO films. The films were grown with different addition times of HYA during the first CBD or without HYA. The individual film thicknesses of the films grown in the first and in the subsequent CBD steps were deduced from cross section SEM micrographs.

	Film thickness in nm (±10 nm)	
Time of HYA addition (first CBD)	After first CBD	After second CBD

0 min	<10	320
15 min	60	375
30 min	80	365
45 min	120	460
no addition	120	630

Obviously, the thickness of the films grown during the first CBD step varies strongly with the addition time of HYA: The later the HYA is added, the thicker the film grows during this step. The thickness increased from <10 nm for films prepared with immediate HYA addition to 120 nm when HYA was added after 45 min. Notably, the film grown without HYA also displays a thickness of 120 nm, corroborating the finding that the growth of the ZnO film has already ceased at this point in time. We conclude that the addition of HYA during the first growth step strongly suppresses the growth of ZnO perpendicular to the support.

The thickness of the films grown during the second CBD step on the layers formed in the first CBD also follow a particular trend, that is, the films deposited during the first CBD step influence the thickness of the films grown during the second CBD step. The earlier the HYA is added during the first CBD step, the thinner the films obtained after the second CBD grow. The ZnO film thicknesses increased from 320 nm (for films which were prepared with immediate addition of HYA during the first CBD) to 460 nm (when the addition took place only after 45 min). The film grown on the substrate prepared during the first CBD without HYA displays the largest thickness of about 630 nm. This also demonstrates that the films obtained in the first CBD strongly influence the further ZnO deposition.

The transmittance of the films is not influenced by the addition of HYA. For films prepared with and without HYA addition, average transmittances of approximately 80% were observed in the visible range.

**Figure 7:**
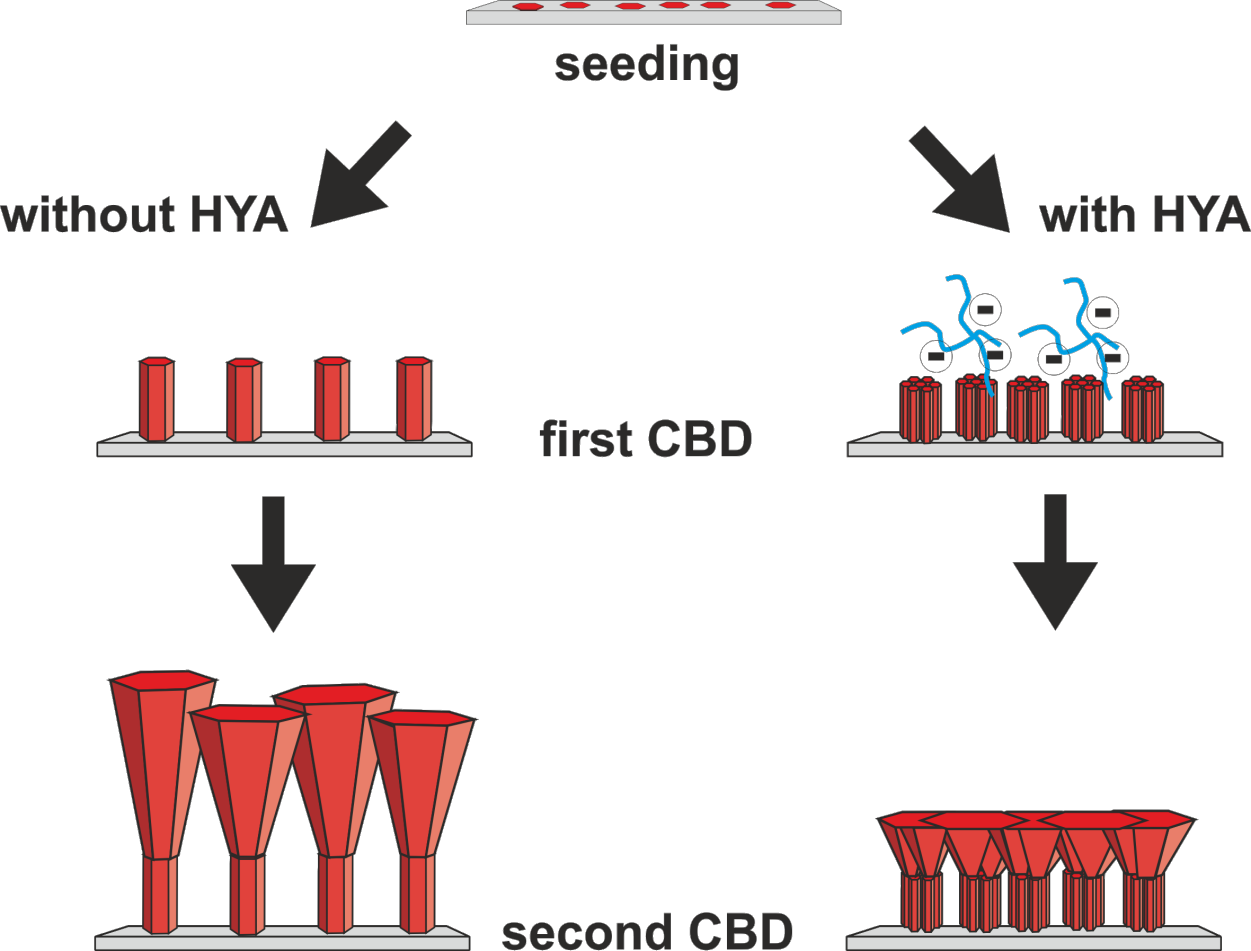
Scheme of the proposed mechanism for the three-step ZnO film deposition process described in this work.

Combining the results from XRD and FE-SEM investigations to form a cohesive theory, we propose the following mechanism for the film formation, as illustrated in Figure 7. First, the seeds deposited during the first step support the growth of ZnO. On such seeds, an array of highly vertically aligned ZnO nanorods grows under CBD conditions as previously reported [15,17,43–44]. Notably, these nanorods do not overlap. Thus, although the thickness of such a nanorod array is quite large (120 nm), the actual mass deposited (as inferred from the intensity of the XRD signal) is rather small. During the subsequent CBD process following the protocol of Baxter and Schmuttenmaer [48], the Zn^2+^ ion concentration is drastically increased. In addition to ongoing axial growth, lateral growth of the ZnO nanorods is also supported. Thus, they grow together and form a dense layer on top of the deposited film. Compared to the films reported by Baxter and Schmuttenmaer, our films prepared in the presence of HYA appear more dense and regular at the surface. When HYA is added during the first CBD step, vertical growth is hindered. This effect is more noticeable when HYA is immediately added and such a film has a thickness of less than 10 nm. When HYA was added at 15 or 30 min after the start of the CBD step, the film thickness increased to 60 or 80 nm, respectively. However, according to the XRD intensity, much more ZnO is deposited in these cases. This can be explained by an enhanced lateral growth of the nanorod bundles onto the support during the first CBD step, as revealed by the SEM images in Figure 4. The earlier the HYA is added during this step, the more the individual nanorods overlap. These differences in the films then lead to different growth characteristics in the subsequent CBD process. In general, the films become thinner and are more strongly intergrown after the final CBD when HYA was present in the first CBD. We surmise that due to the enhanced lateral deposition of ZnO in the first CBD step, more supporting surface area for further ZnO growth during the final CDB is available. This surface area is finely structured as it is based on bundles of thin zincite crystallites; each of the latter could possibly serve as nucleation centers for crystal deposition during the subsequent CBD steps. Thus, the bundled ZnO rods, which have preferably grown laterally during the first CBD step, allow the formation of a more dense ZnO layer during the final CBD. Consequently, as the total material supply is limited, axial growth is diminished, that is, the films become thinner.

### Electrical properties of the films

ZnO is a semiconductor with a direct bandgap of 3.37 eV [19]. At room temperature and without light illumination, ZnO provides only very few charge carriers in the conduction band leading to a moderate electrical conductivity [49]. The conductivity of ZnO dramatically increases when it is exposed to a light source.

Improved conductivity can also be achieved via doping of ZnO [50–52], which is not a topic covered in this work. Here, we use electrical conductivity data as an additional means to judge the quality of the films. In order to obtain reproducible and meaningful results, and to be able to compare the quality of our films, the electrical properties were determined under UV irradiation corresponding to the bandgap energy (370 nm). The values of the sheet resistance as well as the specific resistance of completely processed ZnO films after the second CBD are listed in Table 2. The sheet resistance of our films was above 1 kΩ/sq and the sheet resistance of the samples which were only seeded was larger than 100 MΩ/sq.

**Table 2:** Electrical properties of ZnO films: comparison of the specific resistance and sheet resistance of ZnO films after the second CBD step. The films were grown with different addition times of HYA during the first CBD step and without HYA. The films were exposed to UV irradiation during the measurement.

Time of HYA addition [min]	Specific resistance [Ω∙cm]	Sheet resistance [kΩ/sq]

0	0.09	3.1 ± 0.7
15	0.08	1.9 ± 0.5
30	0.29	6.4 ± 0.7
45	0.17	2.7 ± 0.4
no addition	0.86	11.5 ± 0.7

The film prepared without addition of HYA yielded a sheet resistance of 11.5 kΩ/sq, or normalized to its thickness of ≈750 nm, a specific resistance of 0.86 Ω∙cm results. All films prepared with HYA showed lower sheet resistances than the unmodified film, regardless of the time when HYA was added. The lowest sheet resistances with values of 1.9 and 2.7 kΩ/sq were obtained for fully processed films when HYA was added after 15 or 45 min during the first CBD step. The films prepared with an early addition of HYA are much thinner, as was previously explained in detail. Consequently, the films grown with immediate HYA addition or with HYA addition after 15 min displayed small specific resistances of 0.09 and 0.08 Ω∙cm. For comparable films (e.g, undoped ZnO films prepared via CBD methods), specific resistances of 0.25 Ω∙cm [48] and 0.648 Ω∙cm [53] have been reported for as-grown and annealed films, respectively. We therefore claim that the use of the biological additive hyaluronic acid can improve the electrical conductivity and the general quality of zinc oxide films grown with CBD processes.

## Conclusion

This study describes a three-step deposition process of ZnO films from solution at low temperature. The process consists of a seeding step and two subsequent CBD steps. During the first CBD step, hyaluronic acid (a natural polysaccharide) is added. The time of the addition strongly influences the morphology of the deposited ZnO. The ZnO structure can be tailored from individual rods to finer structures consisting of bundles of rods [42]. The HYA suppresses the ZnO growth perpendicular to the support but enhances the lateral deposition of ZnO. In general, the earlier the HYA is added during the first CBD step, the finer the crystallites appear and the denser and thinner the films grow. The films grown under the influence of HYA during the first CBD step were used as supports for the third step – an additional CBD process where the films were “sealed”. The film thickness and the degree of intergrowth after this CBD step strongly depend on the morphology of the support obtained after the first CBD step. In general, films which are denser and more finely structured after the first CBD lead to thinner and more strongly intergrown layers in the final CBD. Both findings can be linked to the availability of more nucleation sites on the finer-structured and denser support.

The fully processed ZnO films deposited under the influence of HYA show significantly lower film sheet resistance and specific resistance as compared with ZnO films prepared without additives. These lower specific resistances are most probably a result of enhanced crystal domain intergrowth caused by mediation of the deposition by hyaluronic acid. The introduction of this naturally occurring polysaccharide thus enhances the quality of chemical bath-deposited zinc oxide films. This opens up further possibilities for the use of natural polymers such as polysaccharides for the preparation of technologically relevant materials and devices. In bio-inspired synthetic approaches, such polymers can act in a similar way as in biomineralization processes, influencing the growth and controlling the morphology and arrangement of the resulting crystallites.

## Experimental

### Synthesis

All experiments were performed with micropore-filtered water (Clear UV, SG Wasseraufbereitung und Regenerierungsstation GmbH, Hamburg; maximum conductivity of 0.055 µS/cm). The films were prepared on glass slides in three steps according to Figure 1.

First step: seeding. The crystal precursors were deposited on glass slides using a protocol according to Greene and co-workers [43]. For this purpose, the glass slides were immersed in a 5 mM zinc diacetate dihydrate (reagent grade, Aldrich) ethanolic solution for 10 sec, then cleaned with ethanol. This procedure was repeated five times. Afterwards the films were annealed at 350 °C for 20 min. The whole procedure was repeated once [43].

Second step: first CBD. The deposition of ZnO on the seeded glass slides was performed in 100 mL screw cap bottles containing 0.75 g zinc dinitrate hexahydrate (purum, Aldrich) and 0.35 g hexamethylentetramine (HMTA, puriss, Aldrich) dissolved in 75 mL water. The mixture was vigorously stirred until a nearly clear solution was obtained. The seeded glass slides were immersed into this solution and fixed in a vertical position by using a holding device machined from Teflon. This corresponds to the start of the time measurement. The reaction was initiated by rapid heating to 90 °C while gently stirring. 83 mg of hyaluronic acid (HYA, sodium salt from *Streptococcus equi*, MW ≈1600 kDa, Aldrich) was dissolved in 25 mL of water under vigorous stirring and added to the solution described above after a certain time (0, 15, 30 or 45 min). The amount of HYA added corresponds to a molar ratio of (1/12):1 with regard to the repeating unit of HYA (*M* = 0.4013 kg/mol) and the Zn(II) ion concentration. The combined solutions were kept at 90 °C for one hour in total. Afterwards, the glass slides were taken out of the screw cap bottle, rinsed with water, carefully washed with ethanol in an ultrasonic bath, and dried at 60 °C.

Third step: second CBD. The second CBD step was performed according to the reaction conditions reported by Baxter and Schmuttenmaer [48]. 2.97 g of zinc dinitrate hexahydrate (purum, Aldrich) and 1.405 g hexamethylentetramine (HMTA, puriss, Aldrich) were dissolved in 100 mL of water under vigorous stirring in a screw cap bottle until an almost clear solution was obtained. The glass slides treated according to step 1 and 2 were dipped into this solution and vertically arranged by a Teflon holder. The reaction was initiated by heating the screw cap bottle rapidly to 85 °C under gentle stirring. After one hour, the glass slides were removed from the screw cap bottle, rinsed with water, carefully washed with ethanol in an ultrasonic bath, and dried at 60 °C.

### Characterization

X-ray diffraction patterns were recorded on a STOE (Darmstadt, Germany) Theta-Theta diffractometer in reflection geometry using monochromatic, Cu Kα radiation. SEM micrographs were taken on a JEOL (Tokyo, Japan) 6700F FE-SEM operating at an acceleration voltage of 2 kV and a working distance of 3 mm. For electron microscopy analysis, the glass slides with ZnO were properly cut and fixed with silver paste (Plano GmbH, Wetzlar, Germany) onto a copper block. The average film thicknesses were determined with ImageJ 1.43 software based on cross section FE-SEM micrographs by measurement of at least four different locations. UV–vis transmission measurements were performed on a Cary 5E spectrometer (Varian Inc., Palo Alto, USA) in order to determine the optical transparency of the ZnO films. To ensure that only the transmittance of the ZnO films was measured, the spectrum of a cleaned glass slide was used for a background correction. The contact angle measurement of the ZnO films was carried out using a Surftens apparatus (OEG GmbH, Frankfurt, Germany). The electrical conductivity measurements were performed with a 2100 Multimeter (Keithley Instruments Inc., Cleveland, USA). For the conductivity measurement, the films were contacted by 2 parallel lines of silver paste (Plano GmbH, Wetzlar, Germany) of 1 cm in length and with 1 cm distance between them. The sheet resistance of the fully processed films was recorded under UV irradiation (370 nm, 8 W power). The specific resistance values were calculated as a product of the sheet resistance with the thickness of the corresponding film.
